# Ex vivo conditioning of peripheral blood mononuclear cells of diabetic patients promotes vasculogenic wound healing

**DOI:** 10.1002/sctm.20-0309

**Published:** 2021-02-18

**Authors:** Rica Tanaka, Rie Ito‐Hirano, Satoshi Fujimura, Kayo Arita, Hiroko Hagiwara, Tomoya Mita, Masayoshi Itoh, Hideya Kawaji, Takasuke Ogawa, Hirotaka Watada, Haruchika Masuda, Takayuki Asahara, Hiroshi Mizuno

**Affiliations:** ^1^ Division of Regenerative Therapy Juntendo University Graduate School of Medicine Tokyo Japan; ^2^ Department of Plastic and Reconstructive Surgery Juntendo University School of Medicine Tokyo Japan; ^3^ Intractable Disease Research Center Juntendo University Graduate School of Medicine Tokyo Japan; ^4^ Center for Genomic and Regenerative Medicine Juntendo University Graduate School of Medicine Tokyo Japan; ^5^ Department of Metabolism & Endocrinology Juntendo University Graduate School of Medicine Tokyo Japan; ^6^ RIKEN Preventive Medicine and Diagnosis Innovation Program Wako Japan; ^7^ Preventive Medicine and Applied Genomics Unit RIKEN Center for Integrative Medical Sciences Yokohama Japan; ^8^ Tokyo Metropolitan Institute of Medical Science Tokyo Japan; ^9^ Department of Dermatology Juntendo University School of Medicine Tokyo Japan; ^10^ Department of Basic Clinical Science, Division of Regenerative Medicine Tokai University School of Medicine Isehara Japan

**Keywords:** diabetes mellitus, mononuclear cell therapy, peripheral blood mononuclear cells, quantity and quality control culture technique, vasculogenesis, wound healing

## Abstract

The quality and quantity of endothelial progenitor cells (EPCs) are impaired in patients with diabetes mellitus patients, leading to reduced tissue repair during autologous EPC therapy. This study aimed to address the limitations of the previously described serum‐free Quantity and Quality Control Culture System (QQc) using CD34+ cells by investigating the therapeutic potential of a novel mononuclear cell (MNC)‐QQ. MNCs were isolated from 50 mL of peripheral blood of patients with diabetes mellitus and healthy volunteers (n = 13 each) and subjected to QQc for 7 days in serum‐free expansion media with VEGF, Flt‐3 ligand, TPO, IL‐6, and SCF. The vascular regeneration capability of MNC‐QQ cells pre‐ or post‐QQc was evaluated with an EPC colony‐forming assay, FACS, EPC culture, tube formation assay, and quantitative real time PCR. For in vivo assessment, 1 × 10^4^ pre‐ and post‐MNC‐QQc cells from diabetic donors were injected into a murine wound‐healing model using Balb/c nude mice. The percentage of wound closure and angio‐vasculogenesis was then assessed. This study revealed vasculogenic, anti‐inflammatory, and wound‐healing effects of MNC‐QQ therapy in both in vitro and in vivo models. This system addresses the low efficiency and efficacy of the current naïve MNC therapy for wound‐healing in diabetic patients. As this technique requires a simple blood draw, isolation, and peripheral blood MNC suspension culture for only a week, it can be used as a simple and effective outpatient‐based vascular and regenerative therapy for patients with diabetes mellitus.

## Graphical abstract

Mononuclear cells (MNCs) were isolated from peripheral blood of diabetic patients and were subjected to a serum‐free Quantity and Quality Control Culture System. Both in vitro and in vivo models revealed vasculogenic, anti‐inflammatory, and wound‐healing effects of MNC‐QQ therapy. The approach can be used as a simple and effective outpatient‐based vascular and regenerative therapy for diabetic patients.

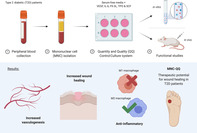

**Significance statement**

Mononuclear cells (MNCs) isolated from humans are used for vascular regenerative therapy. Such therapies are useful for treating non‐healing wounds of patients with diabetes. But isolating large number of functional vasculogenic cells from such patients is a difficult process. In the current study, a new and more practical serum‐free quantity‐control culture method is described. This new technique can restore the functions of human peripheral blood MNCs (PbMNCs) derived from patients with diabetes. PbMNCs derived with this technique showed high vasculogenic, anti‐inflammatory, and wound healing potential in diabetic patients. This is one of the first preclinical studies for vascular regenerative therapy using blood from the patients with diabetes.

## 1 INTRODUCTION

Since the successful isolation of endothelial progenitor cells (EPCs) from peripheral blood in 1997,^1^ autologous mononuclear cells (MNCs) have been derived from bone marrow (BM)^2^, ^3^ and used for clinical vascular regenerative therapy. Autologous BM and peripheral blood MNC (PbMNC) therapy with functional EPCs are clinically effective in ischemia and have been used successfully in nondiabetic individuals.^2^, ^4^ Furthermore, autologous granulocyte‐colony stimulating factor (G‐CSF)‐mobilized peripheral blood CD34+ cell therapy is safe, feasible, and effective in diabetic patients with chronic non‐healing ulcers.^5^ However, clinical trials of autologous cell therapy with freshly isolated CD34+ cells have limitations in the isolation and expansion of cells. The number and functional capacity of EPCs (CD34+ cells) are severely impaired in diabetic patients, limiting their clinical value for autologous EPC therapy.^5^, ^6^, ^7^, ^8^ Since vasculogenic cells, such as CD34+ or CD133+ cells, constitute a very small percentage of PbMNCs (0.01%) and BM‐MNCs (0.1%), a large amount of BM aspiration or peripheral blood apheresis and injection of G‐CSF is needed for cell therapy applications.^5^ Additionally, PbMNCs are heterogenous and may include cells such as B cells, Killer T cells, NK cells, and M1 macrophages, which can cause inflammatory side effects of cell therapy.^9^, ^10^ Ameliorating functional deficits and increasing cell counts to a sufficient level are, thus, central to successful autologous EPC therapy in diabetic patients.^11^ Therefore, isolation techniques with a simple methodology and minimal effort are needed. We identified a serum‐free Quantity and Control culture method (QQc) that could restore the functionality of BM‐derived murine diabetic c‐kit+Sca‐1+lin− (KSL) cells and peripheral blood CD34+ cells of patients with diabetes.^12^ In subsequent study, we observed post QQc treatment, human peripheral blood CD34+ cells demonstrated significantly high vasculogenic and wound healing potency in mouse model.^13^ However, isolating clinically sufficient number of CD34 + cells from small amount of human peripheral blood is difficult as apheresis can be a large burden for the patients. Therefore, we aimed to develop a method to accumulate highly regenerative cells from a simple cell isolation technique.

A novel QQc for PbMNCs obtained from serum‐free expansion media with five cytokines enhanced the vasculogenic potential of EPCs derived from healthy subjects and facilitated the preparation of PbMNCs for regenerative phenotype activation.^14^, ^15^, ^16^ Without any requirement of isolation of CD34+ cells, this new technique is more practical than CD34‐QQ cell therapy. In this study, we hypothesized that QQc would restore the functions of human PbMNCs derived from patients with diabetes and therapy with the MNC‐QQ cells (monocytes, CD34+, and lymphocytes) would demonstrate vasculogenic, anti‐inflammatory, and wound‐healing potential in diabetic patients with ischemic disease and non‐healing wounds. Leveraging the prominent clinical potential of PbMNC‐QQ, this is one of the first pre‐clinical studies for vascular regenerative therapy using blood from patients with diabetes.

## 2 METHODS

### 2.1 Ethics

All participants provided informed consent. This study was conducted in complete accordance with the Declaration of Helsinki and was approved by Juntendo University's ethics committee and review board. The study followed the recommendations in the Guide for the Care and Use of Laboratory Animals of the National Institutes of Health and was approved by the guidelines of the Institutional Animal Care and Use Committee of Juntendo University School of Medicine (Tokyo, Japan) based on the Guide for the Care and Use of Laboratory Animals (National Research Council).

### 2.2 Participants

Patients with type 2 diabetes were selected from the outpatient department of Juntendo University Hospital, Tokyo, Japan. Nonsmoking males, between 20 and 80 years of age with HbA_1C_ < 8.0 g/dL were included. Patients with severe heart failure, hemodialysis, peritoneal dialysis, infectious disease, hematologic disease, inflammatory disease, or malignant tumors were excluded. Healthy volunteers were recruited to serve as controls (Table 1).

**TABLE 1:** Summary of profiles of patients with diabetes and healthy subjects

	Patients with diabetes	Healthy subjects
N	36	13
Age (years)	63.4 ± 5.4	61.2 ± 4.4
BMI	24.5 ± 4.2	21.2 ± 2.6
DM duration (years)	23.1 ± 14.4	N/A
HbA_1c_ (%)	6.5 ± 1.1	N/A
Systolic BP (mmHg)	129.3 ± 21.2	N/A
Diastolic BP (mmHg)	73.6 ± 12.7	N/A
Total Cholesterol (mg/dL)	150.8 ± 28.0	N/A
HDL (mg/dL)	50.2 ± 17.8	N/A
LDL (mg/dL)	88.8 ± 24.1	N/A
Triglyceride (mg/dL)	101.0 ± 47.3	N/A

*Notes:* Values are expressed as the mean ± SD. N/A: not applicable. No significant differences observed for age (*P* > .05). BMI: DM vs Healthy, *P* = .0055.

Abbreviations: BMI, body mass index; BP, blood pressure; DM, diabetes mellitus; HbA_1c_, glycated hemoglobin; HDL, high density lipoprotein; LDL, low density lipoprotein.

### 2.3 Preparation of PbMNCs

Peripheral blood from healthy volunteers and patients with diabetes mellitus (DM) was obtained with written informed consent under the institutional approval of the Clinical Investigation Committee of Juntendo University School of Medicine and Tokai University School of Medicine. The preparation of PbMNCs was performed as described previously.^14^ Peripheral blood samples (50 mL/subject) were drawn into tubes containing EDTA‐2Na (Venoject II, Terumo, Tokyo, Japan). The whole blood was placed into 50‐mL tubes and centrifuged (400*g*, 25°C, 10 minutes). PbMNCs were prepared from the collected buffy coat suspended with EDTA‐PBS by density gradient centrifugation (400*g*, 25°C, 30 minutes) using Histopaque‐1077 (Sigma, St. Louis, Missouri). After washing with EDTA‐PBS, the cells were treated with NH_4_Cl solution (pH 7.3), washed twice with ETDA‐PBS, and, finally, suspended in an appropriate medium or ETDA‐PBS.

### 2.4 Ex vivo expansion culture

PbMNCs prepared from healthy donors and patients with DM were processed in an ex vivo serum‐free expansion culture system named QQc, as described previosuly.^17^ For this, fresh PbMNCs (pre‐QQc) were seeded at a density of 2 × 10^6^ cells/well in six‐well Primaria plates (BD Falcon, Franklin Lakes, New Jersey) with 2 mL/well of Stemline II medium (Sigma, St. Lois, Missouri) supplemented with recombinant human vascular endothelial growth factor (rhVEGF; 50 ng/mL), rh interleukin‐6 (rhIL‐6; 20 ng/mL), rh Fms‐related tyrosine kinase‐3 ligand (rhFlt‐3L; 100 ng/mL), rh thrombopoietin (rhTPO 20 ng/mL), rh stem cell factor (rhSCF; 100 ng/mL) (all from PeproTech, Rocky Hill, New Jersey), and an antibiotic cocktail (Invitrogen), and cultured for 7 days at 37°C in a 5% CO_2_ atmosphere. After 7 days, without subculture or re‐feeding, post‐QQc cells were harvested by gently pipetting and washing the wells with EDTA‐PBS, and were then suspended in an appropriate medium.

### 2.5 RNA Seq

MNCs were lysed in RLT solution (Qiagen, Germany) and total RNA was prepared following the manufacturer's protocol. All RNA‐seq was performed using a TruSeq Stranded RNA‐seq kit (Illumina) following the manufacturer's protocol. The ribosomal RNA depletion method was followed using a Ribo‐Zero kit (Illumina) or NEBNext rRNA depletion kit for samples smaller than 10 ng. All libraries were applied on a HiSeq2500 sequencer for 50 paired‐end reads in the high throughput mode with HiSeq Flow Cell v3. The obtained reads were aligned with the reference sequence of the human genome, GRCh37, using STAR.^18^ The alignments with a mapping quality of more than 20 were counted per gene of Gencode V19^19^ using feature counts.^20^ Gene expression levels were quantified as a logarithm of CPM (counts per million) base 2 through RLE normalization using edgeR.^21^ Hierarchical clustering of gene expression was estimated by hclust in R, with the complete linkage method based on the distance of 1‐Pearson's correlation coefficient.

### 2.6 EPC colony‐forming assay

Cells were placed in an EPC‐colony‐forming assay (CFA) semisolid culture medium, MethoCult SF H4236 (Stem Cell Tech.), supplemented with rhVEGF (50 ng/mL), rhFGF‐2 (50 ng/mL), rhSCF (100 ng/mL), rhIGF‐1 (50 ng/mL), rhIL‐3 (20 ng/mL), rhEGF (50 ng/mL) (PeproTech), 30% FBS (CCB, Nichirei Biosci., Tokyo, Japan), heparin (2 IU/mL) (Ajinomoto, Tokyo, Japan), and an antibiotic cocktail. EPC‐CFA working medium was diluted with 30% FBS‐IMDM, and 1 mL of suspended PbMNCs was seeded with blunt‐end needles (Nipro, Osaka, Japan) attached to 1‐mL syringes into a 35‐mm Primaria culture dish to promote cell adhesion (2 × 10^5^ cells/dish). On day 16, the number of adherent colonies per dish was measured using a gridded scoring dish (Nunc, Thermo Fisher Scientific) using a phase‐contrast light microscope (Eclipse Ti‐U; Nikon, Tokyo, Japan). Based on the maturation stage of colony‐forming cells, EPCs were classified as primitive EPC colony‐forming units (pEPC‐CFUs) or definitive EPC (dEPC)‐CFUs.

### 2.7 EPC culture assay

PbMNCs and PbMNCs‐QQ were suspended in EGM‐2MV medium containing 5% FBS and depleted hydrocortisone (1 × 10^6^ cells/mL), and were cultured in a human fibronectin‐coated 96‐well Primaria plate (1 × 10^5^ cells/100 μL/well) for 7 days. On day 7, the medium was replaced with fresh medium containing FITC‐labeled *Ulex europaeus* agglutinin I (FITC‐UEA‐I, 10 μg/mL, Vector Lab., Burlingame, California) and 1,1′‐dioctadecyl‐3,3,3′,3′‐tetramethylindo‐carbocyanine perchlorate‐labeled acetylated low‐density lipoprotein (DiI‐acLDL, 10 μg/mL, Biomedical Tech., Stoughton, Massachusetts). The attached cells were cultured for 4 hours for EPC labeling. The cells were washed with PBS twice and fixed with 4% paraformaldehyde phosphate buffer solution (PFA, Wako, Osaka, Japan) at 4°C for 30 minutes. The wells were then covered by Vector shield with DAPI (Vector) to avoid fluorescence decay and nuclear staining. The plates were kept at 4°C in the dark prior to observation. In each well, three to five micrographs with random fields were acquired using a fluorescence microscope (Keyence, Osaka, Japan). The FITC‐UEA‐1 and DiI‐acLDL double‐positive cells were counted as early EPC.

### 2.8 Tube formation assay

The tube formation assay was performed as described previously.^17^ Briefly, PbMNCs were labeled with low‐density lipoprotein from human plasma, acetylated DiI complex (DiI‐Ac‐LDL) (Biomedical Technologies, Inc) at 37°C for 2 hours. The 96‐well plates were pre‐coated with 50 μL/well of Biocoat Matrigel (Corning). The gels were then overlaid with 1 × 10^3^ pre‐ and post‐QQc cells and cocultured with 1.5 × 10^4^ human umbilical vein endothelial cells (HUVECs,; Lonza, Basel, Switzerland) in 50 μL of EBM‐2 containing 2% FBS (Lonza) and incubated at 37°C in a 5% CO_2_ atmosphere for 4 hours. Wells containing only HUVECs were used as controls. The wells were photographed using a phase‐contrast microscope. Total DiI‐Ac‐LDL‐labeled cells incorporated into the tubes were analyzed using fluorescence microscopy (BZ‐9000; Keyence, Osaka, Japan).

### 2.9 FACS analysis

Pre‐ or post‐QQc cells in EDTA‐PBS containing 2% FBS (FACS buffer) were treated with FcR blocking reagent (Miltenyi, Auburn, California) at 4°C for 30 minutes, and then stained with the specific antibodies PE/Cy7‐labeled anti‐CD31 (clone: WM59, BioLegend, San Diego, California), PE‐labeled anti‐CD34 (clone: 581, BioLegend), APC‐labeled anti‐CD184 (CXCR4, clone: 12G5, BD Bioscience, San Jose, California), APC‐labeled anti‐CD133 (clone: AC133, Miltenyi), Alexa Fluor‐700‐labeled anti‐CD3+ (clone: UCHT1, BioLegend), APC/Cy7‐labeled anti‐CD14 (clone: HCD14, BioLegend), PE/Cy7‐labeled anti‐CD206 (MMR, clone: 15‐2, BioLegend), PerCP/Cy5.5‐labeled anti‐CCR2 (CD192, clone: K036C2, BioLegend), BV421‐labeled anti‐CD127 (clone: A019D5, BioLegend), BV421‐labeled anti‐CD8 (clone: RPA‐T8, BioLegend), FITC‐labeled anti‐CD4 (clone: RPA‐T4, BioLegend), PE‐labeled anti‐CD25 (Clone: M‐A251, BioLegend), or proper isotype controls for each color at 4°C for 30 minutes. The cells were washed twice with FACS buffer then analyzed using an FACS Aria III flow cytometer (BD Franklin Lakes, New Jersey). The data were processed using FlowJo software (Tree Star, Ashland, Oregon) and the scattergrams were gated using three different cell sizes, with the smallest cells in gate A indicating the lymphocyte area, the middle cell size in gate B indicating monocytes, and the large cell size in gate C (Supporting Information Figure S1). The absolute cell number was calculated by multiplying the FACS % of each cell type with the total cell number of 1 × 10^7^ cells collected from PbMNCs (pre‐QQ cells) and post‐QQ cells.

### 2.10 Quantitative real time PCR

Pre‐ and post‐QQc PbMNCs were lysed in RLT solution (Qiagen, Germany) and total RNA was prepared following the manufacturer's protocol. Complimentary DNA (cDNA) was synthesized from the total RNA using Superscript II (Invitrogen) and was used as a template for PCR. PCR was carried out using cDNA mixed with TaqMan fast universal PCR master mix and TaqMan probes on an ABI 7500 fast real‐time PCR system. TaqMan probes used were angiopoietin (Ang)‐1 (Hs00181613_m1), Ang‐2 (Hs00169867_m1), insulin‐like growth factor (IGF)‐1 (Hs01547656_m1), leptin (Hs00174877_m1), vascular endothelial growth factor (VEGF)‐A (Hs99999070_m1), VEGF‐B (Hs00173634_m1), hepatocyte growth factor (HGF) (Hs00300159_m1), IL‐8 (Hs00174103_m1), IL‐10 (Hs00961622_m1), matrix metalloprotease (MMP)‐2 (Hs01548727), MMP‐9 (Hs00234579_m1), IL1‐β (Hs01555410_m1), TNF‐α (Hs00174128_m1), TGF‐β (Hs0098133_m1), FGF‐2 (Hs00266645_m1), and 18S‐rRNA (4308329) (all from Applied Biosystems). The data were analyzed as delta (Δ) CT (target CT‐18S‐rRNA CT) and demonstrated as 2^−ΔCT^.

### 2.11 Angiogenesis array

The levels of expressed angiogenesis‐related proteins in the secretion from MNC‐QQ cells were studied. Supernatants from the MNC‐QQ culture were analyzed using a Luminex cytokine multiplex analysis kit (R&D Systems, Catalog # LXSAMSM). Briefly, 7 days after MNC‐QQ culture, the MNC‐QQ culture supernatant were collected and centrifuged at 200*g* for 5 minutes. Afterward, all samples were filtered through a Millex 0.22‐μm membrane to remove debris and stored at −80°C until testing. The samples were removed for multiplex analysis according to the manufacturer's recommendations. Using Stemline II Hematopoietic Stem Cell (SL2) as a control in QQ media supplemented with five cytokines (5G), multiplex analysis was performed using a Luminex 100/200 instrument and the resulting data were interpreted using proprietary data analysis software.

### 2.12 Wound‐healing model

To evaluate cell function in vivo, pre‐ and post‐QQc MNCs from diabetic donors were transplanted into a mouse wound‐healing model. Following the methods of previous studies,^13^ 8‐10‐week‐old male BALB/c nude mice (CAnN.Cg‐Foxn1nu/Crlj; Charles River Laboratories Japan, Inc, Tokyo, Japan) and 50 mg/kg streptozotocin‐induced diabetic mice (Sigma‐Aldrich, St. Louis, Missouri) were used. Mice were considered diabetic if they maintained glucose levels above 300 mg/dL for at least 4 weeks before wounding. The mice were anesthetized and depilated, then two excisions were made on the dorsum bilateral skin using a biopsy punch (6 mm, Kai Industries, Gife, Japan). The excisions were in full‐thickness, including the hypodermis and panniculus carnosus. The wound edges were marked with India ink tattoos. Silicon stents with an 8‐mm internal diameter were sutured with 5‐0 nylon around each wound to minimize skin contracture and ensure healing by secondary intention. On the third day post‐operation, each wound was injected with either PBS as a control or cells from one of the PbMNC (1 × 10^4^ cells/wound) groups from the patients: pre‐QQc healthy control, pre‐QQc DM, post‐QQc healthy control, and post‐QQc DM. The wounds in the mice were photographed and digitally measured (BZ II Analyzer application software, BZ‐H2A, Keyence). The percent wound closure was measured photogrammetrically on days 0, 3, 7, 10, and 14, using the following formula:

$$\left(1–\left[\left(\text{wound area}\;\mathrm{on}\;\text{examination}\;\mathrm{day}\right)/\left(\text{wound area}\;\mathrm{at}\;\mathrm{day}\;0\right)\right]\times 100\right).$$

### 2.13 Histology and immunofluorescence

Wounds were harvested from euthanized mice on days 7 and 14 with a full‐thickness excision that extended 3 mm radially past the original wound edge and were demarcated with India ink. The harvested wounded skin samples were dissected for paraffin or frozen sectioning. Sections of 5 μm thickness were cut from the central region of the wound. They were then deparaffinized and rehydrated before staining with hematoxylin‐eosin to measure epithelial and granulation thicknesses using fluorescence microscopy (BZ‐9000). Epithelial thickness included the distance from the surface to the basic membrane of the epithelium. Granulation thickness was measured by the distance from the base of the epithelium to the surface of the panniculus carnosus layer.

For the detection of vascular regeneration, paraffin sections were stained with rat anti‐mouse CD31 antibody (clone: MEC13.3, BD) as an endothelial marker and developed with 3,3‐diaminobenzidine (DAB, Vector Laboratories, Burlingame, California). The CD31+ vessels per field were counted at 200× magnification.

Wound maturity was quantified using the van Gieson staining protocol. Paraffin sections were processed with staining solution as described previously.^22^ Images were digitalized (BZ‐9000) and analyzed using Adobe Photoshop CS6 (Adobe Systems, San Jose, California).

To follow cell trafficking of the adoptively transferred MNC‐QQ, the frozen tissues were stained with mouse anti‐human mitochondrial antibody using a rabbit anti‐mouse kit (Discovery MoMap kit, 760‐137, Ventana Medical Systems, Oro Valley, Arizona) and CD31+ antibody (clone: MEC13.3, BD). Tissue sections were mounted using a mounting medium containing DAPI. Slides were digitally captured, and the double‐positive vessels were counted at 40× and 20× magnifications using a confocal microscope (Olympus FV1000D IX81, SPD; Olympus, Tokyo, Japan).

### 2.14 Statistical analysis

A Wilcoxon signed‐rank test (paired nonparametric test) was performed to compare in vitro assay results between the pre‐ and post‐QQc groups. A Mann‐Whitney test (non‐paired nonparametric test) was performed to compare in vivo results between healthy and DM groups, as well as in vivo data comparisons for different donors. All analyses were performed using GraphPad Prism 5. Statistical significance was defined as *P* < .05. Data are presented as the mean ± SD.

## 3 RESULTS

### 3.1 Healthy and DM PbMNC numbers post‐QQc

**FIGURE 1:**
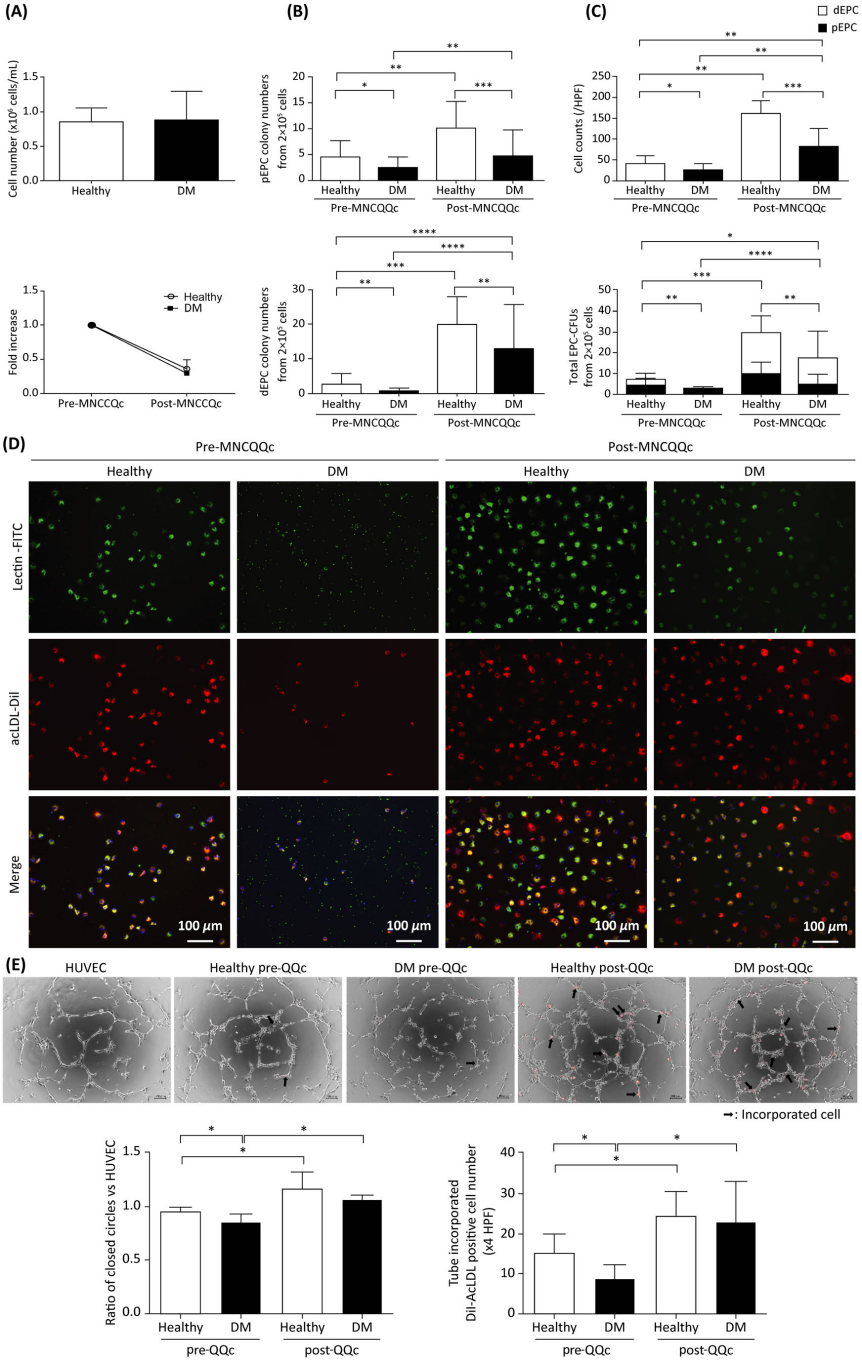
Flow cytometry analysis of peripheral blood mononuclear cell (PbMNC) and endothelial progenitor cell (EPC) subpopulation numbers and tube formation promotion pre‐ and post‐Quantity and Quality Control Culture System (QQc) treatment. Cumulative quantitative graphs of, A, the number of peripheral PbMNCs, B, the number of pEPC and dEPC colonies, and, C, the number of EPCs in healthy and diabetic cultures (n = 13). D, Representative images of early EPCs captured with a fluorescence microscope (scale bar = 100 μm). E, Promotion of tubular formation by pre‐ and post‐MNC‐QQ compared to untreated HUVEC controls. Representative images (scale bar = 100 μm) and cumulative quantitative graphs of a tube formation assay showing the number of tubules formed in each group and DiI‐acLDL incorporation into HUVEC‐formed tubes in each group *P < .05, **P < .005, ***P < .0005. dEPC, definitive EPC; DM, diabetic; MNC‐QQc, mononuclear cell treated with quality‐quantity culture; PBS, phosphate‐buffered saline; pEPC, primitive EPC

The numbers of fresh PbMNC were 85.1 ± 20.3 × 10^4^ cells/mL in healthy subjects (n = 13) and 89.2 ± 40.3 × 10^4^ cells/mL in patients with DM (n = 13), respectively. Following QQc, the number of PbMNCs decreased to 17.1 ± 4.9 × 10^4^ cells/mL (0.36 ± 0.13‐fold) in the healthy group and 14.6 ± 7.6 × 10^4^ cells/mL (0.28 ± 0.83‐fold) in the DM group, respectively. There were no significant differences in the numbers of PbMNCs between healthy and DM subjects before or after QQc (Figure 1A).

### 3.2 Significant difference in the vasculogenic potential of PbMNCs post‐QQ

Pre‐QQc cells from healthy donors had a larger number of pEPC‐CFUs (4.51 ± 3.20 vs 2.49 ± 2.01; *P* < .05) and dEPC‐CFUs (1.83 ± 7.95 vs 0.75 ± 0.78; *P* < .01) compared to patients with DM. Pre‐QQc cells from healthy donors responded better to QQc in terms of total colony number, with dEPC‐CFUs showing a remarkable increase (1.83 ± 7.95 vs 19.74 ± 7.99; *P* < .0001). Though the increases in pEPC‐CFUs and dEPC‐CFUs were lower in the PbMNCs of patients with DM compared to those in healthy subjects, the DM PbMNCs also showed an increase in total colony number after QQc (3.24 ± 2.37 vs 17.62 ± 15.90; *P* < .0001). The total colony numbers of the DM group (especially dEPC‐CFU) post‐QQc MNCs were higher than those of healthy pre‐QQc MNCs (Figure 1B).

EPC culture assay results showed that the number of early EPCs was lower in patients with DM (27 ± 14 vs 41 ± 18 cells/HPF; *P* < .05). Early EPCs increased significantly in both the healthy control group (41 ± 18 vs 161 ± 31 cells/HPF; *P* < .01) and the DM group after QQc (27 ± 13 vs 84 ± 42 cells/HPF; *P* < .001) (Figure 1C,D).

### 3.3 Promotion of tube formation upon culture with post‐QQc PbMNCs

Pre‐QQc PbMNCs of patients with DM showed reduced tube formation compared to HUVEC control without cells. Post‐QQc PbMNCs promoted tube formation in HUVECs cocultured for 4 hours. There was no significant difference in cells between the healthy and diabetic groups post‐QQc PbMNCs (Figure 1E).

#### 3.3.1 QQ affects anti‐inflammatory angiogenic cell populations in healthy donors and patients with DM

**FIGURE 2:**
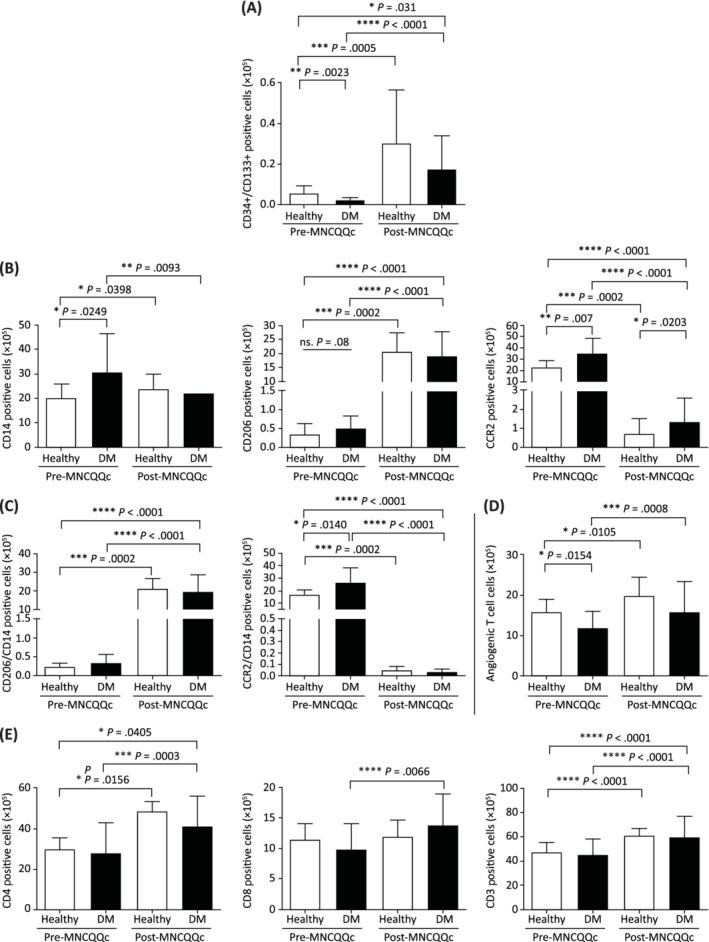
The effect of Quantity and Quality Control Culture System (QQc) treatment on certain PbMNC populations in healthy volunteers and patients with diabetes mellitus (DM). Cumulative representative graphs of flow cytometry analysis of cell populations in healthy vs DM whole cells presented as, A, the absolute number of positivity of CD34+/CD133+ stem cell markers, B, the absolute number of positivity of CD206+, CD14+, and CCR2+ macrophage markers, C, the absolute number of positivity of CD206+/CD14+ and CCR2+/CD14+; D,E, angiogenic and T cell markers: CD3+, CD4+, and CD8+; *P < .05, **P < .005, ***P < .0005. DM, diabetic; MNC‐QQc, mononuclear cell treated with quality‐quantity culture; PBS, phosphate‐buffered saline

FACS scattergrams showed an increase in size of the side scatter area and forward scatter area of PbMNCs after QQc culture. The size and appearance of the scattergrams did not differ between healthy and DM groups in either pre‐ or post‐QQc conditions (Supporting Information Figure S1). Post‐QQc culture, DM‐PbMNCs were highly angiogenic and anti‐inflammatory. A significant increase in the number of vascular CD34+/CD133+ stem cells, CD206+ cells, angiogenic T cells, and Tregs was observed. Concomitantly, a significant decrease in the number of immune response‐related cells (CCR2, CD56+, and CD19+) was observed after QQc (Figure 2A‐E) (Supporting Information Table 1).

#### 3.3.2 Significant increases in the expression of genes related to angiogenesis, wound‐healing, and anti‐inflammation after QQc

**FIGURE 3:**
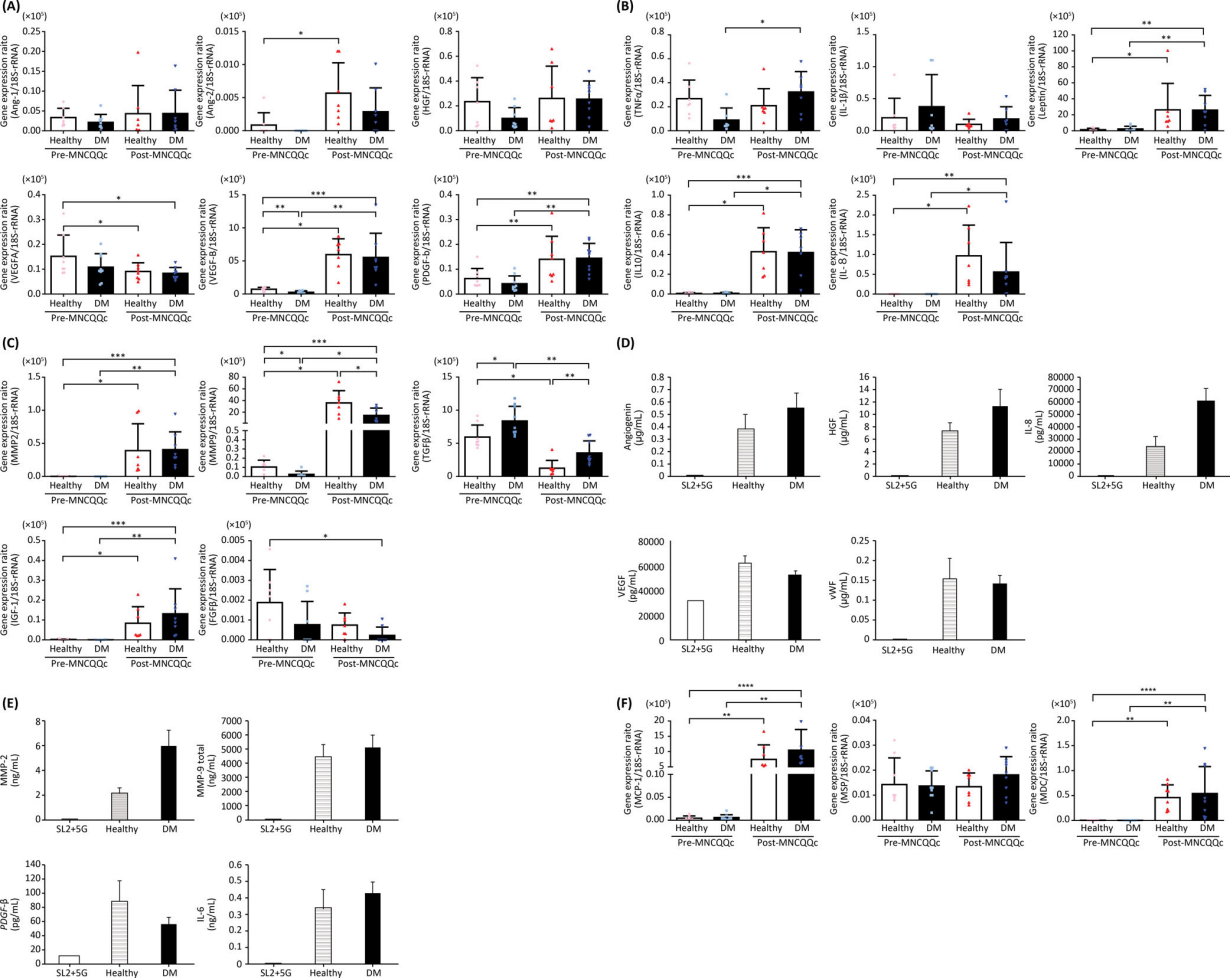
Levels of mRNA and proteins related to angiogenesis, wound‐healing, and anti‐inflammation after Quantity and Quality Control Culture System (QQc) treatment. Levels of mRNA related to, A, angiogenesis, B, anti‐inflammation, and C, wound‐healing measured by quantitative PCR. Levels of proteins related to, D, angiogenesis, E, macrophages, and F, wound‐healing measured by cytokine multiplex analysis. *P < .05, **P < .005. DM, diabetic; MNC‐QQc, mononuclear cell treated with quality‐quantity culture; Sl2 + 5G, Stemline II Hematopoietic Stem Cell +5 cytokines

RNA‐seq was performed to investigate genome‐wide changes in the expression of genes related to angiogenesis, wound‐healing, and anti‐inflammation between pre‐ and post‐QQ cells in patients with DM. Their hierarchical clustering indicated a clear shift in the expression of genes associated with angiogenesis after QQc (Supporting Information Figure S2). RT‐PCR was carried out to confirm the mRNA levels of angiogenesis‐related genes (Supporting Information Table S2). Prior to QQc, the expression of angiogenesis‐related genes, such as *VEGF‐A*, *VEGF‐B*, *Ang‐1*, *Ang‐2*, *PGDF*, and *HGF* (Figure 3A), as well as wound‐healing genes, such as *FGF2* and *IGF‐1* (Figure 3B), were lower in DM PbMNCs compared to those in healthy controls.

Following QQc, the gene expression levels of many pro‐angiogenic growth factors as well as cytokines and wound‐healing genes in DM PbMNCs were significantly increased. *IL‐10*, *IL‐8, Ang‐2*, *IGF‐1*, *MMP2*, and Leptin were expressed only after QQc (Figure 3A‐C). After QQc treatment, in DM PbMNC cell supernatant, the levels of human angiogenesis and macrophage‐related factors angiogenin, HGF, IL‐8, MMP2, MMP 9, IL‐6, MCP‐1, and MDC increased (Figure 3D‐F).

#### 3.3.3 Significant improvement in euglycemic and diabetic wound‐healing following MNC‐QQc diabetic cell therapy

**FIGURE 4:**
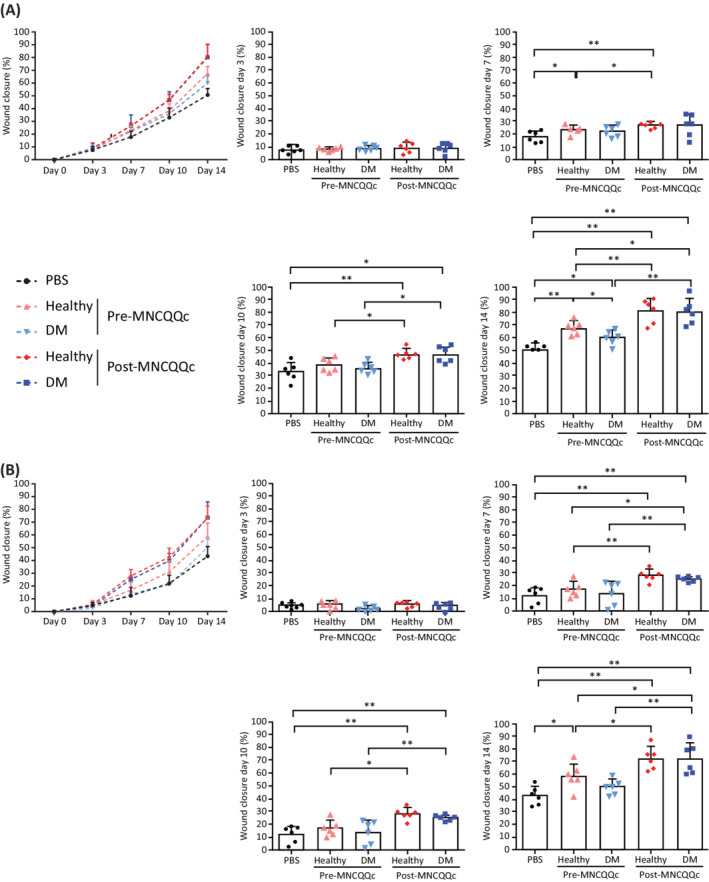
Assessment of wound‐healing time‐points in mice following cell therapy with healthy and diabetic mononuclear cells treated with Quantity and Quality Control Culture System (QQc). The percentage of wound closure was calculated on days 0, 3, 7, 10, and 14 for wounds treated with Pre‐ and post‐MNCs‐QQ from healthy volunteers and diabetic patients in, A, euglycemic mice and, B, diabetic mice. *P < .05, **P < .005. DM, diabetic; MNC‐QQc, mononuclear cell treated with quality‐quantity culture; PBS, phosphate‐buffered saline

The transfer of pre‐QQc diabetic PbMNCs had no effect on the diabetic wounds compared to PBS controls (50.41 ± 6.16% vs 43.35 ± 7.52%, respectively) but had a significant effect on euglycemic wounds compared to PBS controls (60.00 ± 5.66% vs 50.73 ± 5.05%, respectively; *P* < .05) on day 14. Conversely, post‐QQc diabetic PbMNCs elicited significantly faster wound closure than pre‐QQc healthy PbMNCs (79.06 ± 116.5% vs 67.61 ± 5.35%, respectively; *P* < .001) and pre‐QQ diabetic PbMNCs without significant difference between post‐QQc healthy PbMNCs (79.06 ± 11.65% vs 80.99 ± 9.47%, respectively) in euglycemic and diabetic wounds on day 14. However, significant differences in treated pre‐ and post‐QQc healthy groups were observed between euglycemic and diabetic wounds starting from day 7. Differences between treated pre‐ and post‐QQc diabetic wounds was only observed from day 10 onwards (Figure 4).

#### 3.3.4 Improvement in the quality of wound‐healing upon treatment with post‐QQc PbMNCs

**FIGURE 5:**
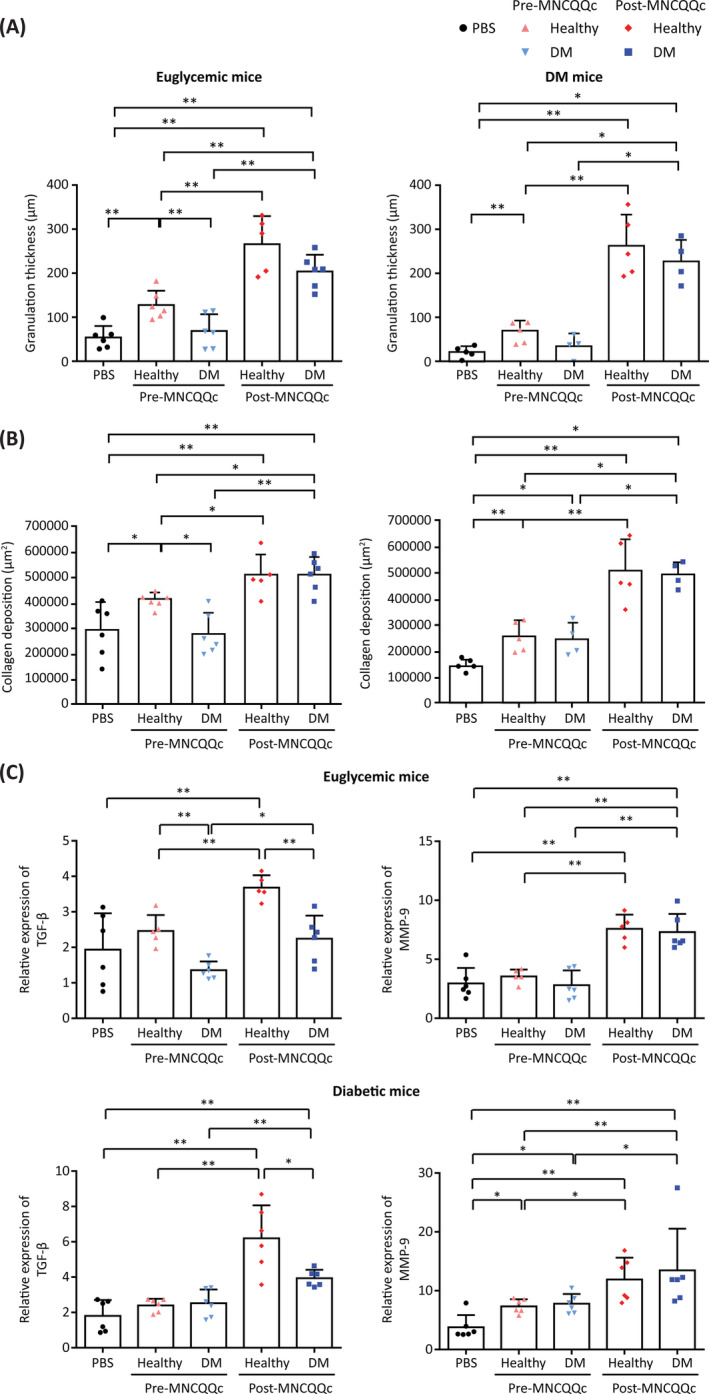
Histological measurement of wound‐healing upon treatment with mononuclear cells treated with Quantity and Quality Control Culture System (QQc). Pre‐ and post‐MNCs‐QQ were transplanted to a mouse wound‐healing model. A, HE granulation thickness of excised wounded skin from day 14 in euthanized mice measured via fluorescence microscopy. B, Wound maturity quantified by the van Gieson staining protocol. C, qRT‐PCR analysis of *TGF‐β* and *MMP‐9* gene expression at day 7, presented as the ratio of mRNA levels of the target gene in a non‐damaged skin sample vs the wounded sample relative to the levels of 18 seconds rRNA. *P < .05, **P < .005. DM, diabetic; MNC‐QQc, mononuclear cell treated with quality‐quantity culture; PBS, phosphate‐buffered saline

Pre‐QQc diabetic PbMNC wounds had significantly lower granulation thickness (68.83 ± 38.08 vs 127.9 ± 32.36 μm; *P* < .05) compared to healthy PbMNCs after 14 days in euglycemic and diabetic wounds (Figure 5A). Post‐QQc‐treatment diabetic PbMNC wounds had significantly increased granulation thickness compared to the pre‐QQc DM PbMNC group in euglycemic (203.8 ± 37.92 vs 68.83 ± 38.08; *P* < .01) and diabetic wounds (229.8 ± 49.82 vs 37.50 ± 26.10; *P* < .05) (Figure 5A, Supporting Information Figure S3). The proportion of mature collagen deposition calculated by van Gieson staining revealed that the wound‐healing potency of diabetic PbMNCs was lower than that of healthy PbMNCs prior to QQc treatment. However, their potency was significantly enhanced after QQc. In fact, it exceeded the potency of healthy PbMNCs and was almost equal to the potency level of post‐QQc healthy PbMNCs (Figure 5B, Supporting Information Figure S4). TGF*‐β* and MMP‐9 mRNA expression levels were significantly upregulated in both euglycemic and diabetic wounds in post‐QQ healthy and diabetic PbMNCs compared to pre‐QQc PbMNCs (Figure 5C).

#### 3.3.5 MNC‐QQc cells increase M2 wound macrophages

We previously reported that diabetic wounds are glucose‐induced inflammatory environments with a high polarization of M1 macrophages but low in M2 macrophages.^23^ Similarly, PBS‐treated diabetic wounds, at day 14, demonstrated high numbers of iNOS‐positive M1 macrophages but low numbers of arginase‐positive M2 macrophages (699.8 ± 66.24 vs 0.11 ± 0.19; *P* < .01). This selective increase in M1 macrophages is possibly owing to the high inflammatory characteristics of diabetic PbMNCs. Following QQc treatment, the number of M1 macrophages significantly decreased whereas that of M2 macrophages significantly increased in both euglycemic and diabetic wounds (Supporting Information Figure S5).

#### 3.3.6 MNC‐QQc enhances the wound vascular density by indirect angiogenesis and direct vasculogenesis in the cell‐transplanted wound

**FIGURE 6:**
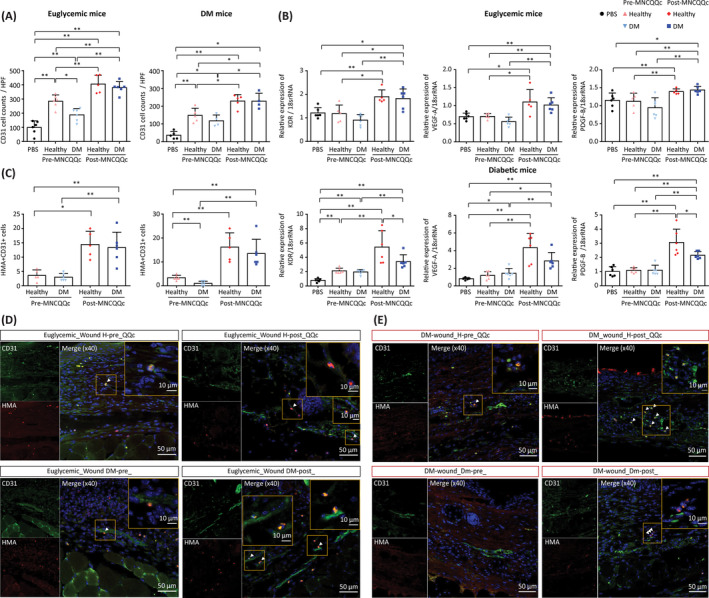
The effect of treatment with MNC‐QQc on wound vascular density by indirect angiogenesis and direct vasculogenesis in the cell‐transplanted wound. A, Vascular density assessed by CD31+ expression. B, Angiogenesis assessed by the expression of *KDR*, *VEGF*, and *PDGFβ* genes at day 7 presented as the ratio of mRNA levels of the target gene in a non‐damaged skin sample vs a wounded sample relative to the levels of 18 seconds rRNA. Frozen tissues were stained with anti‐human mitochondrial antibody and co‐stained with anti‐mouse CD31. C, Direct vasculogenesis assessed by the number of vessels co‐positive for HMA and CD31+. D,E, Representative results of confocal microscopy (scale bar = 50 μm; inset 10 μm). *P < .05, **P < .005. DM, diabetic; MNC‐QQc, mononuclear cell treated with quality‐quantity culture; PBS, phosphate‐buffered saline

Wounds injected with pre‐QQc diabetic PbMNCs demonstrated significantly lower vascular density compared to pre‐QQc healthy PbMNCs in euglycemic (188.0 ± 44.0 vs 283.9 ± 44.97, respectively; *P* < .05) and diabetic wounds (117.5 ± 32.71 vs 148.3 ± 39.77). Although the function of healthy PbMNCs was more pronounced in euglycemic wounds compared to diabetic wounds, post‐QQc PbMNCs of both diabetic and healthy groups showed significantly higher in vivo angiogenic potential by the increase of vascular density in both wounds (Figure 6A).

The post‐QQc diabetic PbMNC‐transplanted wounds demonstrated significantly higher *KDR*, *VEGF*, and *PDGFβ* gene expression on day 7 compared to pre‐QQc diabetic PbMNCs in euglycemic wounds. Similarly, post‐QQc cells showed a potency to promote angiogenesis through pro‐angiogenic growth factor stimulation in the diabetic wounds (Figure 6B).

To assess the direct vasculogenesis of post‐QQc cells, the number of vessels with human mitochondria antigen (HMA)‐positive cells co‐stained with CD31 was assessed. The numbers of HMA‐ and CD31‐positive cells were significantly lower in the wounds transplanted with pre‐QQc diabetic PbMNCs than in those transplanted with post‐QQc diabetic PbMNCs on day 14 in euglycemic (3.0 ± 1.26 vs 13.3 ± 5.35, respectively; *P* < .01) and diabetic wounds (1.0 ± 0.89 vs 13.50 ± 5.95, respectively; *P* < .01). The post‐QQ vasculogenic function of the diabetic PbMNCs was higher than that of pre‐QQc healthy PbMNCs, and not significantly different from the post‐QQc healthy group, demonstrating that diabetic PbMNCs acquires high vasculogenic function through QQc culture (Figure 6C‐E).

## 4 DISCUSSION

Emerging evidence suggests that EPC dysfunction in diabetes leads to poor vasculogenesis and impaired wound regeneration.^24^, ^25^, ^26^ In this study, we hypothesized that a serum‐free QQc culture system would restore the function of PbMNCs in diabetic patients. Successful transplantation of PbMNC‐QQc cells from diabetic patients to mice and subsequent wound‐healing in mice can be clinically translated into cell therapy with the generated MNC‐QQc cells that demonstrate vasculogenic, anti‐inflammatory, and wound‐healing potential in diabetic patients with ischemic disease and non‐healing wounds.

Our results show that PbMNC characteristics in diabetic patients are shifted toward a pro‐inflammatory state in diabetic autologous cell therapy. T cell (CD3+CD4+, CD8+), Treg, and NK cell markers were not significantly different between the two groups, but angiogenic T cell markers were significantly lower, while the B cell marker CD19+ was significantly higher in diabetic patients. This indicates that the PbMNCs of diabetic patients are highly inflammatory and antiangiogenic compared to healthy individuals. Post‐QQ culture, CD34+/CD133+ cells, CD206+ cells, angiogenic T cells, and Tregs significantly increased in diabetic PbMNCs. On the other hand, CCR2+, CD56+, and CD19+ significantly decreased after QQc. These results indicated that under QQc conditions, PbMNCs selectively upregulate the stem cell population for EPCs as well as for anti‐inflammatory pro‐angiogenic monocytes and T lymphocytes, but drastically eliminates the pro‐inflammatory and anti‐regenerative cells.

We developed a serum‐free QQc culture system using PbMNCs that can significantly increase the number of total and differentiated colony‐forming EPCs conditioned for anti‐inflammatory and regenerative phenotypes.^14^ The efficacy of QQc observed in the PbMNCs of diabetic patients was unprecedented. RNA‐sequencing of DM QQ‐PbMNCs was performed to identify whether the gene expression changes were similar to healthy QQ‐PbMNCs prior to investigation with DM QQ‐PbMNCs. Our results confirmed that QQc drastically changed the PbMNC phenotype to pro‐angiogenic and anti‐inflammatory.^14^ This study is the first, to our knowledge, to show that the ex vivo culture of PbMNCs from patients with diabetes develops pro‐angiogenic, anti‐inflammatory, and regenerative functions.

We previously demonstrated that human diabetic CD34+ cells could be expanded ex vivo to restore their impaired functions post‐QQc, as denoted by a markedly higher potential for wound angiogenesis and vasculogenesis compared to pre‐QQc healthy or pre‐QQ DM CD34+ cells.^13^ However, CD34+ cells constitute only 0.01% of peripheral MNCs and are very difficult to isolate. Therefore, for the clinical application of CD34+ cell QQ therapy, the patient must undergo apheresis for PbMNC isolation and magnetic bead‐mediated CD34+ cell isolation, which is physically strenuous. An ideal regenerative therapy would be one where only a small amount of blood needs to be drawn and the cells can be processed ex vivo to generate a population of regenerative cells. There were no significant differences between QQc cells of healthy donors and diabetic patients, demonstrating that the PbMNCs of diabetic patients possess similar potential to healthy MNC‐QQc, but with comparatively higher angiogenic properties. Furthermore, the expression levels of pro‐angiogenic and wound‐healing genes in both healthy and diabetic PbMNCs were upregulated by QQc. The products of these genes possibly act as positive regulators for accelerating vasculogenesis and tissue repair. The total colony numbers of the post‐QQc diabetic MNCs, especially dEPC‐CFUs, were higher than those of the pre‐QQc healthy MNCs, indicating that the vasculogenic potential of diabetic PbMNCs was restored. These results support the idea that pretreating the PbMNCs of diabetic patients prior to cell therapy may enhance the effect of treatment.

M2 macrophages are important in normal and accelerated wound healing.^27^ Diabetic wounds have a predominant population of pro‐inflammatory M1 macrophages, and M2 macrophage cell therapy is effective in wound healing.^23^, ^28^, ^29^ PbMNCs‐QQ contain a high proportion of M2 macrophages as CD206+ cells, which promote an anti‐inflammatory environment within the treated wound. Post‐QQ PbMNCs have a higher tendency to release wound‐healing‐related factors (MMP‐2, MMP‐9, PDGF‐β, and IL‐6), angiogenesis‐related factors (angiogenin, HGF, IL‐8, VEGF‐B, and vWF), and macrophage‐related factors (MCP‐1, MDC, and MSP), suggesting that the roles played by these factors in tissue angiogenesis are the stimulation of wound macrophages for accelerated wound healing and high granulation formation using QQc cells.

In diabetic patients, MNC‐QQc cell therapy accelerated wound closure, maturation, and vascularization in euglycemic and diabetic wounds. QQc enhanced wound healing by upregulating *MMP‐9* and *TGF‐β* gene expression in the cell‐transplanted wound. Post‐cell therapy tissue examination revealed that *TGF‐β* and *MMP‐9* gene expression was significantly higher in both euglycemic and diabetic wounds, suggesting that the wound healing was enhanced by MNCs‐QQc. Furthermore, human DM QQ‐PbMNCs exhibited high potential for direct vasculogenesis in vivo. Wounds transplanted with pre‐QQc diabetic cells showed only a few HMA and CD31+ double‐stained vessels; conversely, wounds transplanted with post‐QQc diabetic PbMNCs showed a significantly higher number of double‐stained vessels, suggesting the acquisition of high vasculogenic potential as a result of QQc. Although wound closure was slightly delayed in diabetic wounds, similar results were observed in all cell therapy groups of diabetic wounds, suggesting the efficacy of QQc cell therapy in a mild diabetic environment. PbMNCs from diabetic foot patients with peripheral arterial disease (PAD) without wounds and diabetic patients with wounds were comparable in terms of cell number, fold‐increase after QQc, vasculogenic potential, cell proliferation, and the presence of cell surface CD34 or CD206 (Supporting Information Figure S6). This observation indicated the clinical utility of autologous PbMNCs to treat diabetic patients with PAD and wounds.

MNC‐QQ is the first vascular regenerative therapy with PbMNCs. It is currently undergoing clinical trials (unpublished). The present study presents the first pre‐clinical trial using blood from diabetic patients. The benefits of the therapy include the ease of blood collection by a simple venipuncture sampling procedure and the simplicity of the isolation process. Limitations of the study, which should be addressed in future work, include small sample sizes, the use of animal models, which have limited clinical relevance to human disease settings, and a limited generalizability of results owing to the narrow patient selection criteria.

## 5 CONCLUSION

This study demonstrates the therapeutic potential of a novel serum‐free QQc culture system using MNCs derived from patients with diabetes. Vasculogenic, anti‐inflammatory, and wound‐healing effects were demonstrated in both in vitro and in vivo models. This system addresses the insufficient efficiency and efficacy of the current naïve MNC therapy for wound‐healing in patients with diabetes. Through this technique, we expect to establish a simple, safe, and effective outpatient‐based vascular and regenerative therapy for diabetic patients.

## CONFLICT OF INTEREST

The authors declared no potential conflicts of interest.

## AUTHOR CONTRIBUTIONS

R.T.: conception and design, financial support, manuscript writing, provision of the study material, collection of data, data analysis and interpretation, approved the manuscript; R.I.‐H., S.F.: collection and assembly of data, data analysis and interpretation, manuscript writing, approved the manuscript; K.A., H.H.: collection and assembly of data, data analysis and interpretation, approved the manuscript; T.M., M.I., H.K., T.O., H.W.: collection and assembly of data, approved the manuscript; H. Masuda, T.A.: advice for conception and design, approved the manuscript; H. Mizuno: administrative support and advice for conception and design, approved the manuscript.

## Supporting information


**FIGURE S1** Scatter diagrams of PbMNCs and QQMNCs in flow cytometry. The thick black lines indicate the cellular‐sized gates of lymphocytes, monocytes, or larger cells.

**FIGURE S2** Expression changes of DM MNCs through QQc. Hierarchical clustering of the expression levels in genes associated with the GO term of angiogenesis (GO:0001525).

**FIGURE S3** Representative photographs of wound sections stained with HE (scale bar: 100 μm, ×100).

**FIGURE S4** Representative photographs of wound sections stained with Van Gieson (scale bar: 100 μm, ×200).

**FIGURE S5** The effect of treatment with MNCs‐QQc on M2 wound macrophages. Frozen wound‐healing tissue samples were stained with antibodies to (A) arginase plus CD68+ and (B) inducible nitric oxide synthase plus CD68+ and were analyzed with confocal microscopy. *P < .05, **P < .005. DM, diabetic; PBS, phosphate‐buffered saline; MNC‐QQc, mononuclear cell treated with quality‐quantity culture.

**FIGURE S6** Number of PBMNCs from diabetic foot patients with peripheral arterial disease (PAD) without wounds and diabetic patients with wounds.

**TABLE S1** Changes in the absolute number of different anti‐inflammatory angiogenic cells pre‐ and post‐QQc treatment.

**TABLE S2** The expression of angiogenesis‐related genes in human peripheral blood mononuclear cells.

## Data Availability

The data that support the findings of this study are available in the Supporting Information of this article.

## References

[1] Asahara T , Murohara T , Sullivan A , et al. Isolation of putative progenitor endothelial cells for angiogenesis. Science. 1997;275(5302):964‐967. 9020076 10.1126/science.275.5302.964

[2] Tateishi‐Yuyama E , Matsubara H , Murohara T , et al. Therapeutic angiogenesis for patients with limb ischaemia by autologous transplantation of bone‐marrow cells: a pilot study and a randomised controlled trial. Lancet. 2002;360(9331):427‐435. 12241713 10.1016/S0140-6736(02)09670-8

[3] Kawamoto A , Asahara T , Losordo DW . Transplantation of endothelial progenitor cells for therapeutic neovascularization. Cardiovasc Radiat Med. 2002;3(3–4):221‐225. 12974378 10.1016/s1522-1865(03)00082-9

[4] Losordo DW , Schatz RA , White CJ , et al. Intramyocardial transplantation of autologous CD34+ stem cells for intractable angina: a phase I/IIa double‐blind, randomized controlled trial. Circulation. 2007;115(25):3165‐3172. 17562958 10.1161/CIRCULATIONAHA.106.687376

[5] Tanaka R , Masuda H , Kato S , et al. Autologous G‐CSF‐mobilized peripheral blood CD34+ cell therapy for diabetic patients with chronic nonhealing ulcer. Cell Transplant. 2014;23(2):167‐179. 23107450 10.3727/096368912X658007

[6] Tanaka R , Wada M , Kwon SM , et al. The effects of flap ischemia on normal and diabetic progenitor cell function. Plast Reconstr Surg. 2008;121(6):1929‐1942. 18520878 10.1097/PRS.0b013e3181715218

[7] Tepper OM , Carr J , Allen RJ Jr , et al. Decreased circulating progenitor cell number and failed mechanisms of stromal cell‐derived factor‐1alpha mediated bone marrow mobilization impair diabetic tissue repair. Diabetes. 2010;59(8):1974‐1983. 20484135 10.2337/db09-0185 PMC2911062

[8] Sawada N , Jiang A , Takizawa F , et al. Endothelial PGC‐1alpha mediates vascular dysfunction in diabetes. Cell Metab. 2014;19(2):246‐258. 24506866 10.1016/j.cmet.2013.12.014 PMC4040246

[9] Kawamoto A , Iwasaki H , Kusano K , et al. CD34‐positive cells exhibit increased potency and safety for therapeutic neovascularization after myocardial infarction compared with total mononuclear cells. Circulation. 2006;114(20):2163‐2169. 17075009 10.1161/CIRCULATIONAHA.106.644518

[10] Sekiguchi H , Ii M , Losordo DW . The relative potency and safety of endothelial progenitor cells and unselected mononuclear cells for recovery from myocardial infarction and ischemia. J Cell Physiol. 2009;219(2):235‐242. 19115244 10.1002/jcp.21672

[11] Jarajapu YP , Grant MB . The promise of cell‐based therapies for diabetic complications: challenges and solutions. Circ Res. 2010;106(5):854‐869. 20299675 10.1161/CIRCRESAHA.109.213140 PMC3816281

[12] Tanaka R , Vaynrub M , Masuda H , et al. Quality‐control culture system restores diabetic endothelial progenitor cell vasculogenesis and accelerates wound closure. Diabetes. 2013;62:3207‐3217. 23670975 10.2337/db12-1621 PMC3749357

[13] Tanaka R , Masuda H , Fujimura S , et al. Quality‐quantity control culture enhances vasculogenesis and wound healing efficacy of human diabetic peripheral blood CD34+ cells. Stem Cells Translational Medicine. 2018;7(5):428‐438. 29573563 10.1002/sctm.17-0043 PMC5905232

[14] Masuda H , Tanaka R , Fujimura S , et al. Vasculogenic conditioning of peripheral blood mononuclear cells promotes endothelial progenitor cell expansion and phenotype transition of anti‐inflammatory macrophage and T lymphocyte to cells with regenerative potential. J Am Heart Assoc. 2014;3(3):e000743. 24965023 10.1161/JAHA.113.000743 PMC4309104

[15] Kado M , Tanaka R , Arita K , et al. Human peripheral blood mononuclear cells enriched in endothelial progenitor cells via quality and quantity controlled culture accelerate vascularization and wound healing in a porcine wound model. Cell Transplant. 2018;27(7):1068‐1079. 29974793 10.1177/0963689718780307 PMC6158547

[16] Ohtake T , Kobayashi S , Slavin S , et al. Human peripheral blood mononuclear cells incubated in vasculogenic conditioning medium dramatically improve ischemia/reperfusion acute kidney injury in mice. Cell Transplant. 2018;27(3):520‐530. 29737200 10.1177/0963689717753186 PMC6038042

[17] Tsukada S , Masuda H , Kwon S , et al. Establishment of vasculogenic colony forming assay in murine endothelial progenitor cells. Circ J. 2007;71(1):269.

[18] Dobin A , Davis CA , Schlesinger F , et al. STAR: ultrafast universal RNA‐seq aligner. Bioinformatics. 2013;29(1):15‐21. 23104886 10.1093/bioinformatics/bts635 PMC3530905

[19] Frankish A , Diekhans M , Ferreira AM , et al. GENCODE reference annotation for the human and mouse genomes. Nucleic Acids Res. 2019;47:D766‐D773. 30357393 10.1093/nar/gky955 PMC6323946

[20] Liao Y , Smyth GK , Shi W . featureCounts: an efficient general purpose program for assigning sequence reads to genomic features. Bioinformatics. 2014;30:923‐930. 24227677 10.1093/bioinformatics/btt656

[21] Robinson MD , McCarthy DJ , Smyth GK . edgeR: a Bioconductor package for differential expression analysis of digital gene expression data. Bioinformatics. 2010;26:139‐140. 19910308 10.1093/bioinformatics/btp616 PMC2796818

[22] Pugazhenthi K , Kapoor M , Clarkson AN , Hall I , Appleton I . Melatonin accelerates the process of wound repair in full‐thickness incisional wounds. J Pineal Res. 2008;44(4):387‐396. 18205728 10.1111/j.1600-079X.2007.00541.x

[23] Shen T , Kanazawa S , Kado M , et al. Interleukin‐6 stimulates Akt and p38 MAPK phosphorylation and fibroblast migration in non‐diabetic but not diabetic mice. PLoS One. 2017;12(5):e0178232. 28542434 10.1371/journal.pone.0178232 PMC5441644

[24] Fadini GP , Albiero M , Vigili de Kreutzenberg S , et al. Diabetes impairs stem cell and proangiogenic cell mobilization in humans. Diabetes Care. 2013;36(4):943‐949. 23111057 10.2337/dc12-1084 PMC3609511

[25] Fadini GP , Agostini C , Avogaro A . Endothelial progenitor cells and vascular biology in diabetes mellitus: current knowledge and future perspectives. Curr Diabetes Rev. 2005;1(1):41‐58. 18220581 10.2174/1573399052952640

[26] Fadini GP , de Kreutzenberg S , Agostini C , et al. Low CD34+ cell count and metabolic syndrome synergistically increase the risk of adverse outcomes. Atherosclerosis. 2009;207(1):213‐219. 19406403 10.1016/j.atherosclerosis.2009.03.040

[27] Kotwal GJ , Chien S . Macrophage differentiation in normal and accelerated wound healing. Results Probl Cell Differ. 2017;62:353‐364. 28455716 10.1007/978-3-319-54090-0_14 PMC5841920

[28] Tellechea A , Bai S , Dangwal S , et al. Topical application of a mast cell stabilizer improves impaired diabetic wound healing. J Invest Dermatol. 2019;S0022‐202X(19):33240‐33243. 10.1016/j.jid.2019.08.449 31568772

[29] Hu MS , Walmsley GG , Barnes LA , et al. Delivery of monocyte lineage cells in a biomimetic scaffold enhances tissue repair. JCI Insight 5. 2017;2(19):e96260. 10.1172/jci.insight.96260 PMC5841872 28978794

