# Supplementary figures and images for: Ex vivo conditioning of peripheral blood mononuclear cells of diabetic patients promotes vasculogenic wound healing
Source: Stem Cells Transl Med. 2021 Feb 18;10(6):895–909. doi: 10.1002/sctm.20-0309 (PMC8133343; doi:10.1002/sctm.20-0309)

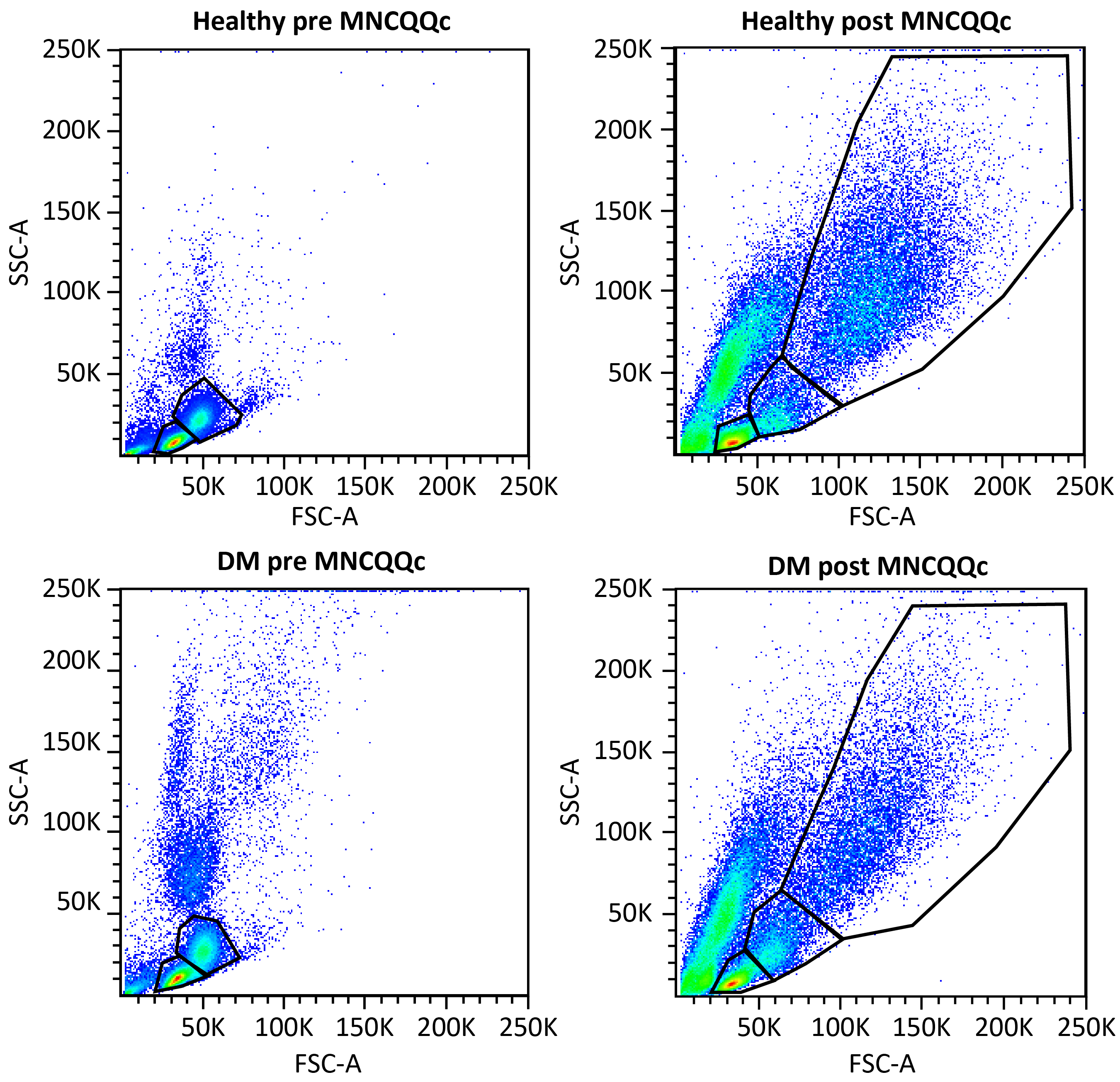

Supplement: Supplementary file 1 — FIGURE S1 Scatter diagrams of PbMNCs and QQMNCs in flow cytometry. The thick black lines indicate the cellular‐sized gates of lymphocytes, monocytes, or larger cells. [file SCT3-10-895-s004.tif]

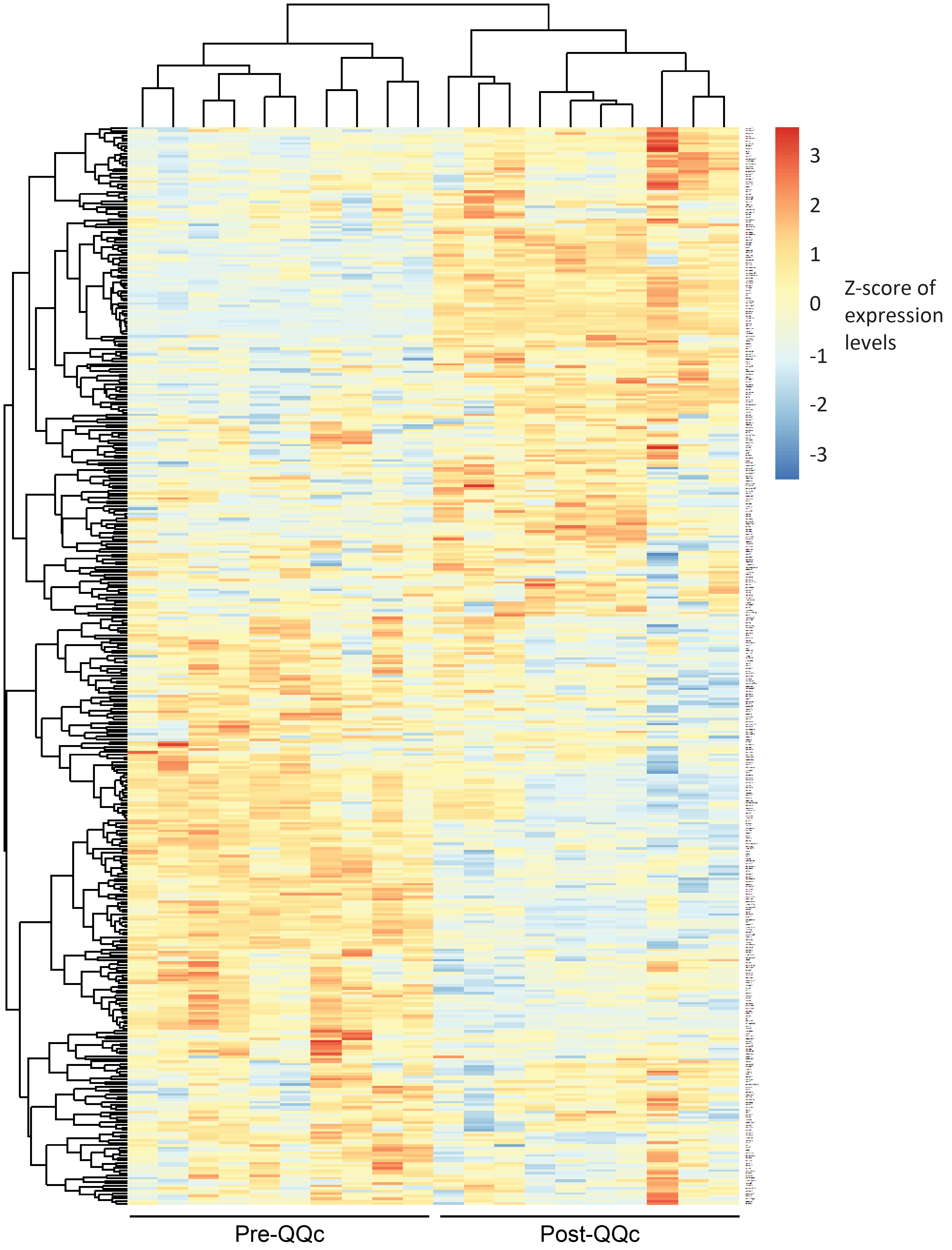

Supplement: Supplementary file 2 — FIGURE S2 Expression changes of DM MNCs through QQc. Hierarchical clustering of the expression levels in genes associated with the GO term of angiogenesis (GO:0001525). [file SCT3-10-895-s003.tif]

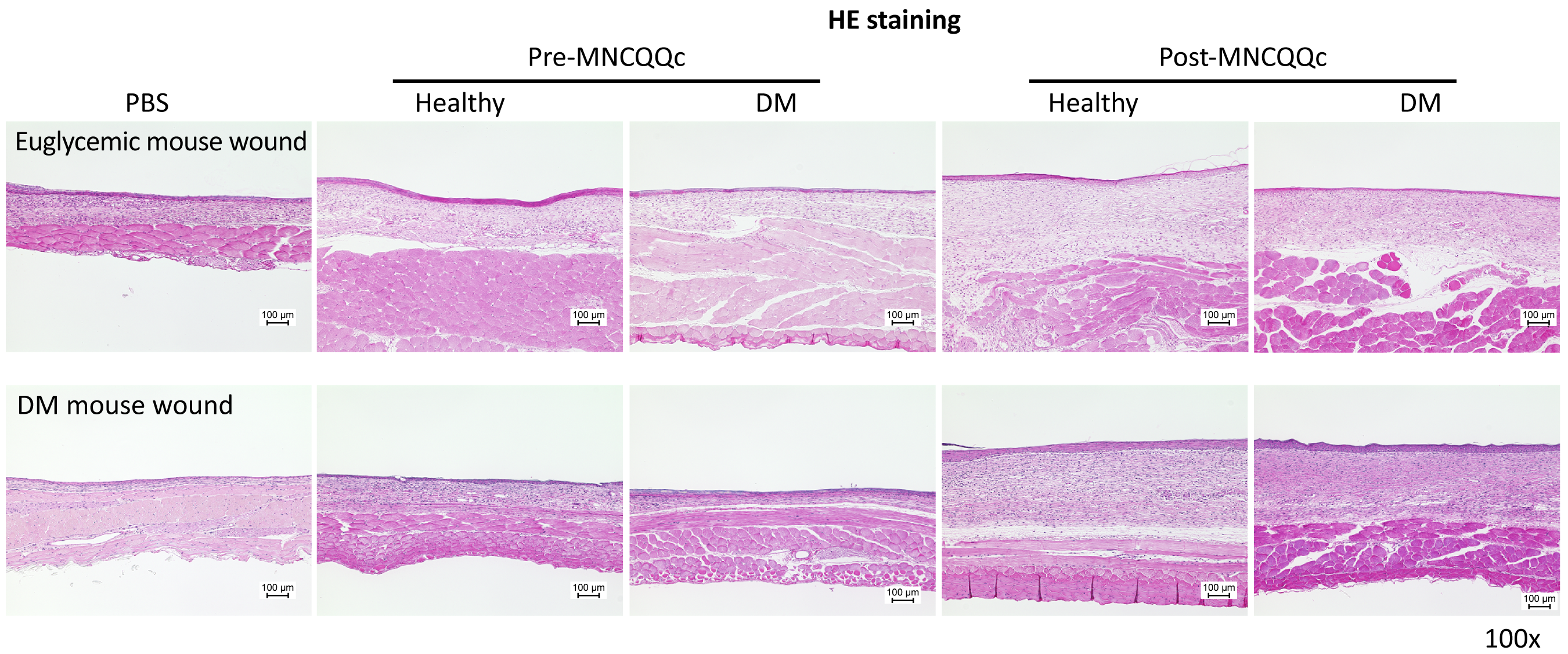

Supplement: Supplementary file 3 — FIGURE S3 Representative photographs of wound sections stained with HE (scale bar: 100 μm, ×100). [file SCT3-10-895-s006.tif]

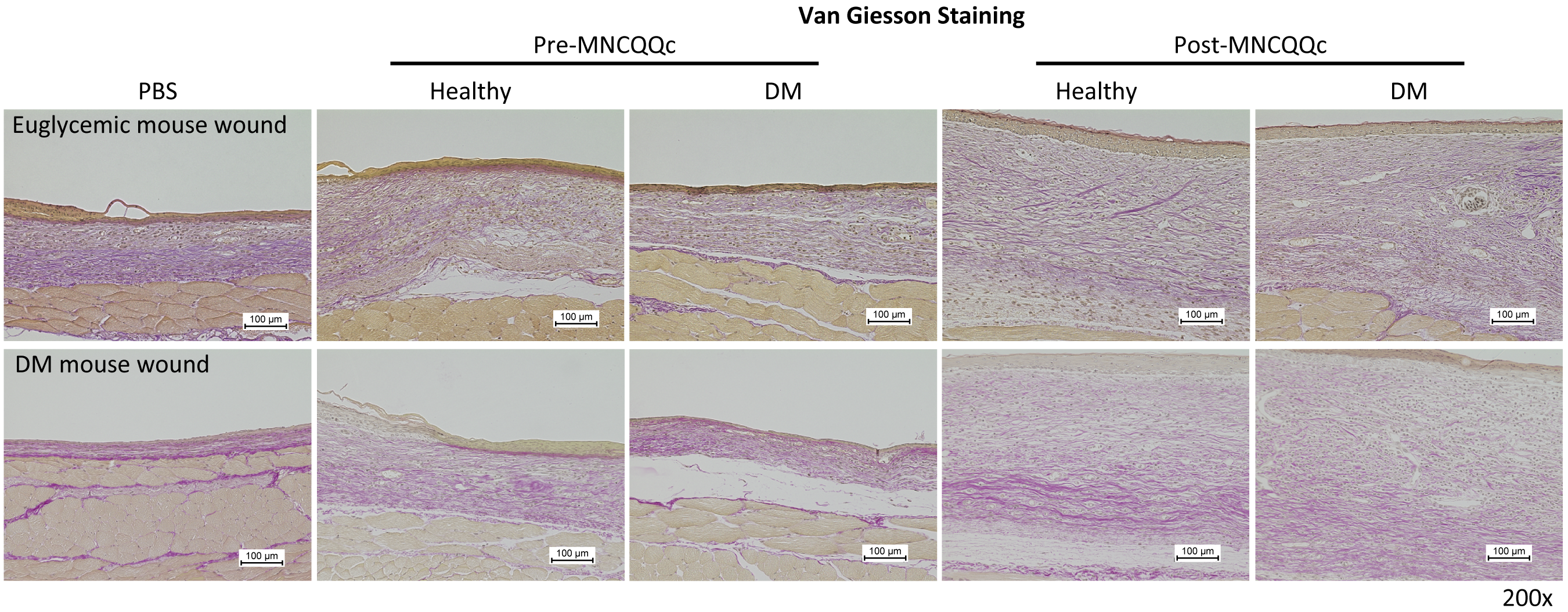

Supplement: Supplementary file 4 — FIGURE S4 Representative photographs of wound sections stained with Van Gieson (scale bar: 100 μm, ×200). [file SCT3-10-895-s002.tif]
