# Supplementary material for: Ex vivo conditioning of peripheral blood mononuclear cells of diabetic patients promotes vasculogenic wound healing
Source: Stem Cells Transl Med. 2021 Feb 18;10(6):895–909. doi: 10.1002/sctm.20-0309 (PMC8133343; doi:10.1002/sctm.20-0309)
Supplement: Supplementary file 5 — FIGURE S5 The effect of treatment with MNCs‐QQc on M2 wound macrophages. Frozen wound‐healing tissue samples were stained with antibodies to (A) arginase plus CD68+ and (B) inducible nitric oxide synthase plus CD68+ and were analyzed with confocal microscopy. *P < .05, **P < .005. DM, diabetic; PBS, phosphate‐buffered saline; MNC‐QQc, mononuclear cell treated with quality‐quantity culture. [file SCT3-10-895-s008.docx]

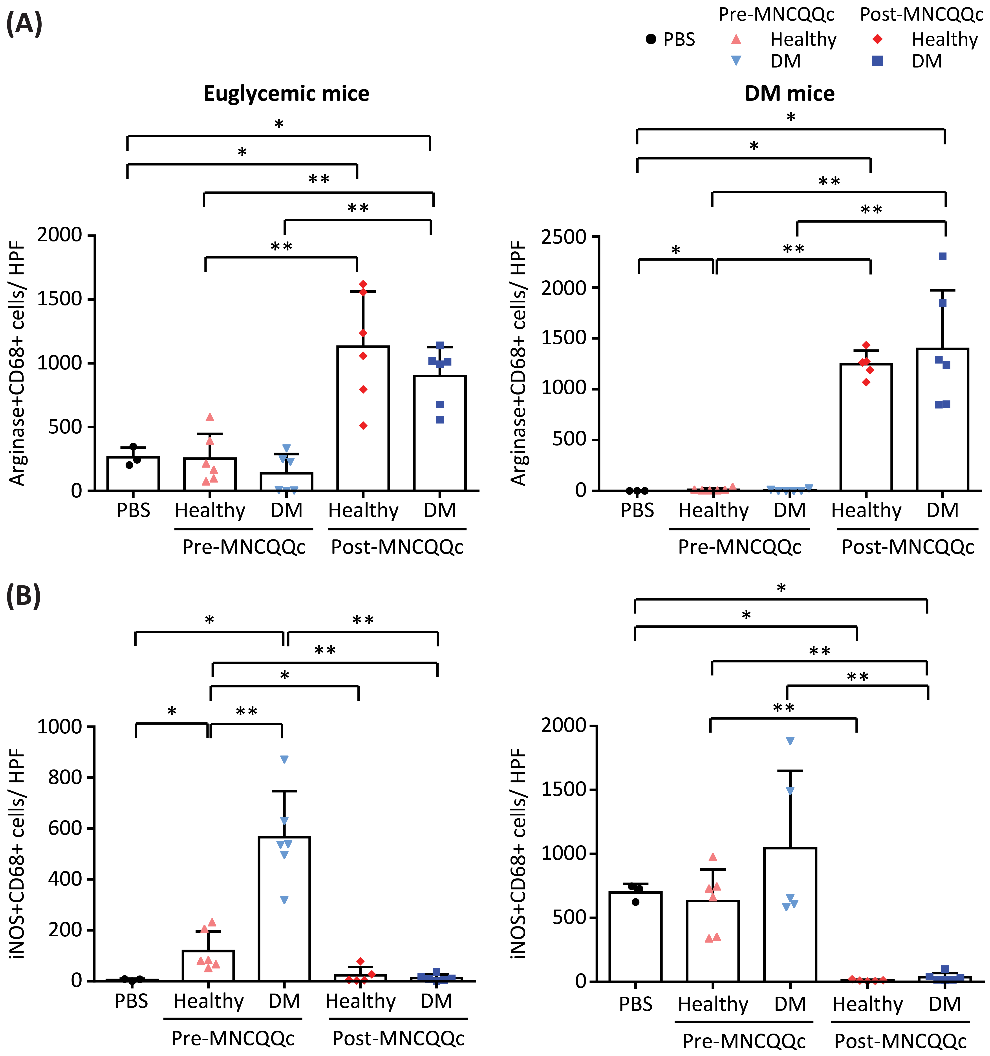


Suppl. Fig. 5. The effect of treatment with MNCs-QQc on M2 wound macrophages. Frozen wound-healing tissue samples were stained with antibodies to (A) arginase plus CD68+ and (B) inducible nitric oxide synthase plus CD68+ and were analyzed with confocal microscopy. *P < 0.05, **P < 0.005. Abbreviations: DM, diabetic; PBS, phosphate‐buffered saline; MNC-QQc, mononuclear cell treated with quality‐quantity culture.
