# Supplementary material for: Ex vivo conditioning of peripheral blood mononuclear cells of diabetic patients promotes vasculogenic wound healing
Source: Stem Cells Transl Med. 2021 Feb 18;10(6):895–909. doi: 10.1002/sctm.20-0309 (PMC8133343; doi:10.1002/sctm.20-0309)
Supplement: Supplementary file 6 — FIGURE S6 Number of PBMNCs from diabetic foot patients with peripheral arterial disease (PAD) without wounds and diabetic patients with wounds. [file SCT3-10-895-s005.docx]

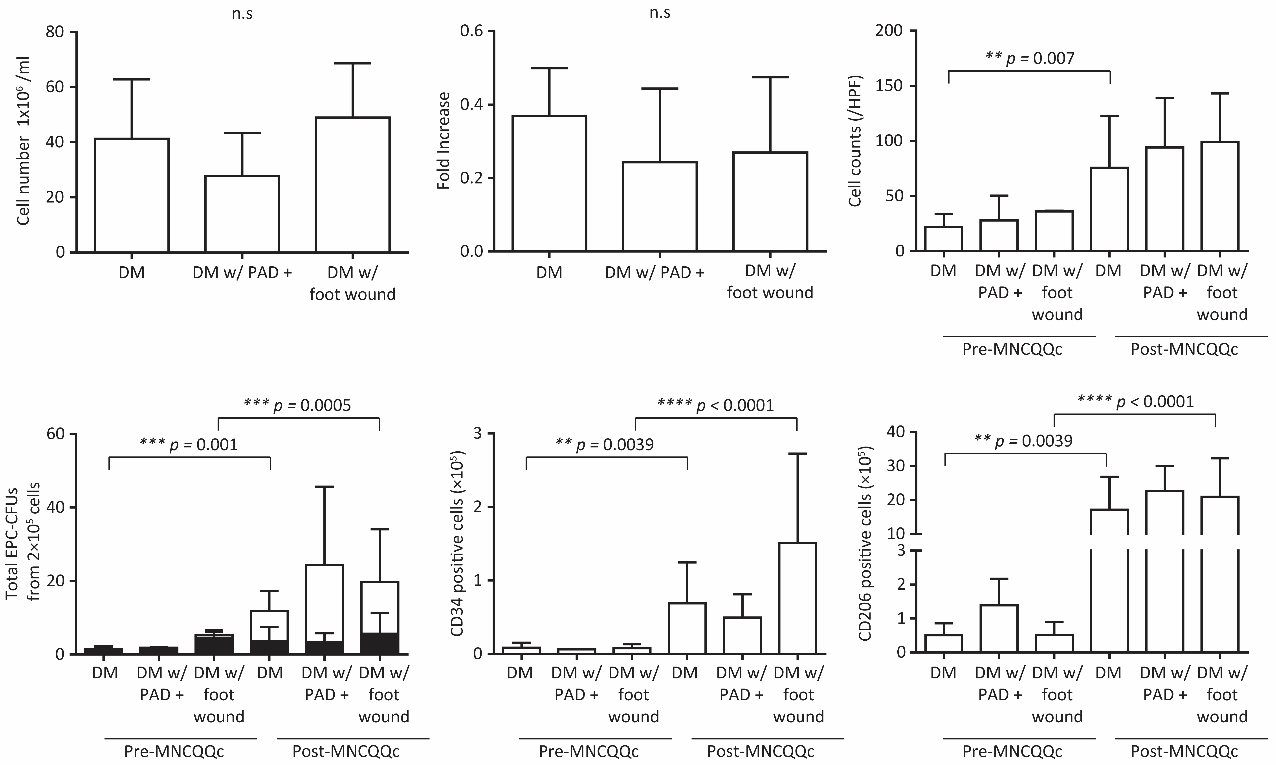


Suppl. Fig. 6. Number of PBMNCs from diabetic foot patients with peripheral arterial disease (PAD) without wounds and diabetic patients with wounds.
