# Supplementary material for: Ex vivo conditioning of peripheral blood mononuclear cells of diabetic patients promotes vasculogenic wound healing
Source: Stem Cells Transl Med. 2021 Feb 18;10(6):895–909. doi: 10.1002/sctm.20-0309 (PMC8133343; doi:10.1002/sctm.20-0309)
Supplement: Supplementary file 7 — TABLE S1 Changes in the absolute number of different anti‐inflammatory angiogenic cells pre‐ and post‐QQc treatment. [file SCT3-10-895-s007.docx]

Supplemental Data

Supplemental Table 1: The absolute number (x10^5^) of different anti-inflammatory angiogenic cells pre- and post-QQc treatment

|  | Pre-MNCQQc | | Post-MNCQQc | |
| --- | --- | --- | --- | --- |
|  | Healthy | DM | Healthy | DM |
| CD34^+^CD133^+^ | 0.05±0.04 | 0.02±0.01 ^++^ | 0.23±0.17 ^***^ | 0.17±0.16 ^****,$^ |
| CD14^+^ | 19.79±5.91 | 30.64±15.59 ^+^ | 23.4±6.55 ^*^ | 21.85±9.43 ^**^ |
| CD206^+^ | 0.32±0.31 | 0.48±0.34 | 20.08±6.94 ^***^ | 18.70±9.15 ^****,$$$$^ |
| CCR2^+^ | 22.53±5.90 | 34.02±14.44 ^++^ | 0.60±0.90 ^***^ | 1.31±1.30 ^****,+,$$$$^ |
| CD206^+^CD14^+^ | 0.18±0.11 | 0.27±0.27 | 20.53±5.95 ^***^ | 19.31±9.43 ^****,$$$$^ |
| CCR2^+^CD14^+^ | 16.80±4.61 | 25.74±12.49 ^+^ | 0.04±0.03 ^***^ | 0.03±0.02 ^****,$$$$^ |
| Angiogenic T | 15.63±3.36 | 11.62±4.34 ^+^ | 19.79±4.53 ^*^ | 15.55±7.84 ^***^ |
| CD3^+^ | 46.69±7.04 | 44.56±13.32 | 60.48±6.33 ^****^ | 58.68±18.26 ^****,$$$$^ |
| CD4^+^ | 29.35±5.88 | 27.21±15.83 | 47.81±5.35 ^*^ | 40.61±15.06 ^***,$^ |
| CD8^+^ | 11.29±2.70 | 9.78±4.29 | 11.92±2.71 | 13.64±5.23 ^**^ |
| CD56^+^ | 18.97±4.37 | 14.21±3.80 | 1.34±2.54 ^*^ | 0.10±0.30 ^**,++,$$$^ |
| Foxp | 1.85±0.34 | 1.26±0.68 | 3.13±0.90 ^*^ | 1.90±1.22 ^**,+^ |
| CD19^+^ | 27.23±3.69 | 39.49±11.30 ⁺ | 5.23±2.35 ^*^ | 2.64±2.29 ^**,$$$^ |

*Pre vs. Post Wilcoxon test

^+^Healthy vs. DM: Mann-Whitney test

^$^ Healthy-pre vs. DM-post

*^,+,$^ *p* < 0.05, **^,++,$$^*p* < 0.01

***^,+++,$SS^ *p* < 0.001,

****^,++++,$$$$^ *p* < 0.0001
