# Supplementary material for: Ex vivo conditioning of peripheral blood mononuclear cells of diabetic patients promotes vasculogenic wound healing
Source: Stem Cells Transl Med. 2021 Feb 18;10(6):895–909. doi: 10.1002/sctm.20-0309 (PMC8133343; doi:10.1002/sctm.20-0309)
Supplement: Supplementary file 8 — TABLE S2 The expression of angiogenesis‐related genes in human peripheral blood mononuclear cells. [file SCT3-10-895-s001.docx]

Table S2: Real-time PCR data for the expression of angiogenesis-related genes in human peripheral blood mononuclear cells

|  | Healthy | | DM | |
| --- | --- | --- | --- | --- |
|  | Pre | Post | Pre | Post |
| FGF2 | 0.0018±0.0016^$^ | 0.00074±0.0006 | 0.00076±0.0117 | 0.00022±0.004 |
| IL-1b | 0.202±0.304 | 0.997±0.078 | 0.376±0.501 | 0.183±0.191 |
| IL-8 | 1.421±1.853 | 26.22±33.16* | 2.38±3.38 | 25.91±18.42**^,$$^ |
| IL-10 | 0.0064±00074 | 0.428±0.242^+^ | 0.0071±0.0091 | 0.419±0.23*^,$$$^ |
| IGF-1 | 0.0023±0.003 | 0.083±0.83 | 0.00055±0.00084 | 0.13±0.125**^,$$$^ |
| Leptin | 0.00±0.00 | 0.967±0.78^+^ | 0.00±0.00 | 0.56±0.75*^,$$^ |
| MMP-2 | 0.022±0.0048 | 0.39±0.41* | 0.00±0.00 | 0.41±0.27**^,$$$^ |
| MMP-9 | 0.103±0.07^+^ | 35.86±20.98* | 0.023±0.036 | 14.25±12.70*^,+,$$$^ |
| TGF-b1 | 5.95±1.80** | 1.25±1.17 | 8.37±2.21^+^ | 3.54±1.82**^,++^ |
| TNF-a | 0.267±0.15 | 0.208±0.14 | 0.089±0.01 | 0.32±0.17* |
| VEGF-A | 0.15±0.086^$^ | 0.091±0.351* | 0.108±0.054 | 0.084±0.023 |
| VEGF-B | 0.722±0.28^++^ | 6.00±2.38* | 0.26±0.22 | 5.53±3.65**^,$$$^ |
| Ang-1 | 0.033±0.023 | 0.043±0.020 | 0.021±0.020 | 0.046±0.059 |
| Ang-2 | 0.00085±0.0018 | 0.0056±0.0045* | 0.00±0.00 | 0.00287±0.0036 |
| HGF | 0.234±0.19 | 0.26±0.26 | 0.099±0.087 | 0.25±0.15 |
| MCP-1 | 0.0045±0.005 | 7.43±4.77** | 0.0058±0.006 | 10.44±6.78**^,$$$$^ |
| PDGF-b | 0.063±0.040 | 0.140±0.093** | 0.042±0.031^+^ | 0.144±0.060**^,$$^ |
| MSP | 0.0142±0.01 | 0.0136±0.006 | 0.0136±0.005 | 0.0181±0.007 |
| MDC | 0.0036±0.002 | 0.458±0.256** | 0.0024±0.001 | 0.541±0.540**^,$$$$^ |

Healthy n = 7~8, DM n = 7~9

The values are presented as the mean ± SD

*Pre vs Post Wilcoxon test

^+^Healthy vs DM: Mann-Whitney test

^$^ Healthy-pre vs. DM-post

*^,+,$^ *p* < 0.05, **^,++,$$^*p* < 0.01

***^,+++, $$$^ *p* < 0.001,

****^,++++,$$$$^*p* < 0.0001
